# SLC25A1-Mediated Cholesterol Accumulation Promotes Endometriosis Progression by Enhancing Endometrial Stromal Cell Proliferation, Invasion, and M2 Macrophage Polarization

**DOI:** 10.7150/ijbs.117146

**Published:** 2026-01-01

**Authors:** Pusheng Yang, Tao Wang, Yaxin Miao, Wenwen Liu, Yiping Zhu, Jing Sun

**Affiliations:** Shanghai Key Laboratory of Maternal Fetal Medicine, Shanghai Institute of Maternal-Fetal Medicine and Gynecologic Oncology, Shanghai First Maternity and Infant Hospital, School of Medicine, Tongji University, Shanghai, 200092, China.

**Keywords:** Endometriosis, cholesterol accumulation, SLC25A1, macrophage polarization.

## Abstract

Endometriosis is an estrogen-dependent chronic inflammatory disorder. Cholesterol (CHO) has been reported to be closely associated with estrogen synthesis and inflammatory responses. Nevertheless, the mechanisms underlying the effects of cholesterol on endometriosis progression and immune response remain to be elucidated. Our research revealed that cholesterol accumulation in ectopic lesions acts as a crucial catalyst for the progression of endometriosis. Using a co-culture system, we simulated a cholesterol-abundant ectopic milieu and demonstrated cholesterol induced M2 macrophage polarization via the STAT6/PPARγ pathway, connecting cholesterol metabolism to immune response in endometriosis. Notably, cholesterol-induced M2 macrophage polarization accelerated the aggressive behavior of ectopic endometrial stromal cells (EESCs). Furthermore, we identified solute carrier family 25 member 1 (SLC25A1) as a pivotal target for regulating cholesterol metabolism in endometriosis, as it significantly upregulated in ectopic lesions and markedly increased intracellular and extracellular cholesterol content. *In vitro* and *in vivo* experiments revealed that cholesterol supplementation reversed the cellular and immune microenvironment alterations caused by SLC25A1 knockdown. Collectively, our results demonstrated that SLC25A1 upregulated the cholesterol metabolism in EESCs and mediated M2 macrophages polarization via the STAT6/PPARγ signaling pathway. Our study on the molecular mechanisms underlying cholesterol accumulation and function may provide potential targets and therapeutic strategies for endometriosis management.

## Introduction

Endometriosis, a highly prevalent gynecological disorder, is characterized by the invasive implantation and progressive proliferation of active endometrial tissues outside the uterine cavity, contributing to dysmenorrhea, chronic pelvic pain, and infertility 1, 2. Endometriosis affects approximately 10 % of women of reproductive age worldwide, adversely compromising the quality of life and mental health of patients 3. Despite advancements in endometriosis therapy, factors such as delayed diagnosis, adverse drug reactions and recurrence continue to impede treatment processes 1, 4, 5. Although the pathogenesis of endometriosis remains unclear, accumulating evidence has indicated that metabolic dysregulation in endometrial stromal cells (ESCs) along with a dysfunctional immune microenvironment are crucial contributors 6-8.

Disrupted cholesterol homeostasis has been considered a crucial factor in malignancies 9, 10. However, the function of cholesterol in endometriosis progression is still unclear. As an estrogen-dependent disease, endometriosis progression is predominantly determined by estrogen concentrations in the local microenvironment 11, 12. Cholesterol (CHO) is an essential precursor to estrogen synthesis 13. It has been reported that ectopic endometrial stromal cells (EESCs) possess a complete aromatase system, resulting in local estrogen accumulation and subsequently EESCs proliferation 14-16. In addition to providing energy for the survival of EESCs, local cholesterol is deeply involved in macrophage polarization and cell signaling. Simultaneously, alterations in the polarization status of macrophages may reciprocally affect cholesterol metabolism 17, 18. However, the mechanisms regulating cholesterol-associated macrophage polarization and endometriosis pathogenesis remain to be elucidated.

Endometriosis is a chronic inflammatory disease characterized by progressive inflammation and immune evasion 19, 20. In endometriosis, macrophages infiltrating ectopic lesions have been demonstrated to enhance the viability and invasiveness of EESCs and play a pivotal role in maintaining homeostasis within the immune microenvironment of endometriosis 21, 22. Macrophages characteristics and phenotypes are determined by their local microenvironment and vary with stage or severity of endometriosis. Accumulating reports indicate that M1 macrophages are gathered in the eutopic endometrium, whereas macrophages in ectopic endometrium tend towards the M2 phenotype 23, 24. In the early stages of endometriosis, macrophages polarize into the M1 phenotype, triggering inflammation and pathogen elimination. As endometriosis progresses, M2 macrophages assume a dominant position and exhibit anti-inflammatory and immunosuppressive features, promoting angiogenesis and tissue repair 25, 26. However, the factors that drive M2 polarization in endometriosis are yet to be explored.

Hence, we explored the relationship between macrophage polarization and cholesterol metabolism in endometriosis. We observed that cholesterol accumulated in ectopic endometrial tissues and promoted the proliferation and invasion of EESCs. Exogenous addition of cholesterol and the supernatants of EESCs was able to polarize macrophages into the M2 phenotype via the STAT6/PPARγ pathway, and this effect was blocked by a cholesterol-depleting agent. In turn, cholesterol-stimulated M2 macrophage polarization promotes the invasive behavior of EESCs. Moreover, we found that SLC25A1 mediates cholesterol accumulation in EESCs. To our knowledge, our study is the first to explore the mechanisms underpinning cholesterol-driven endometriosis progression, establishing a connection with M2 polarization.

## Methods

### Patients and tissues collection

Ectopic endometrial tissues were obtained from 18 patients diagnosed with ovarian endometrial cysts (at stage III/IV, revised American Society for Reproductive Medicine classification of endometriosis, r-AFS) undergoing laparoscopic procedures and histopathological confirmations at the Shanghai First Maternity and Infant Hospital. Control endometrial samples were collected from 18 patients who underwent diagnostic curettage for endometrial polyps, or laparoscopy and hysteroscopy for uterine septum or tubal infertility. The included patients had abstained from hormonal medications for at least three months and underwent surgery during the proliferative phase of the menstrual cycle. The study excluded patients who had acute pelvic inflammatory disease, autoimmune disorders, uterine fibroids or malignant tumors. The clinical details and utilization of the samples are presented in Table S1.

### Cell isolation and culture

Control endometrial stromal cells (CESCs) and EESCs were isolated using type IV collagenase digestion (0.1 %; Sigma, V900891, St Louis, MO, USA) as previously described 27, and resuspended in Dulbecco's Modified Eagle Medium (DMEM)/F12 (Servicebio, G4612, Wuhan, China) containing 10 % fetal bovine serum (FBS) (ScienCell, 0500, Carlsbad, CA, USA) and 1 % penicillin-streptomycin (NCM Biotech, C100C5, Suzhou, China) and incubated at 37 °C in 5 % CO_2_ incubator. To assess the purity of ESCs, the cellular immunofluorescence assay was performed s by staining with cytokeratin and vimentin antibodies, and 3-5 passages of ESCs were used for subsequent experiments. Human endometrial stromal cell (HESC) was obtained from the American Type Culture Collection (ATCC) (CRL-4003, Manassas, VA, USA) and cultured under identical conditions to those of primary ESCs. RAW264.7 cells were cultured in DMEM (Servicebio, G4515, Wuhan, China) (10 % FBS). THP-1 cells (RRID: CVCL_0006) were purchased from the Cell Bank of the Chinese Academy of Sciences (Shanghai, China) and maintained in RPMI-1640 (Servicebio, G4535, Wuhan, China) (10 % FBS). Following treatment with 100 ng/ml phorbol 12-myristate13-acetate (PMA) (MCE, HY-18739, Shanghai, China) for 48 h, THP-1 cells were differentiated and polarized into M0 macrophages. Unless otherwise specified, supernatants were harvested from cells cultured in serum-free medium with the indicated treatments for 48 h at a seeding density of 6 × 10^5^ cells per 10 cm dish.

### Reagents and Treatment

Chemical reagents, including CHO (HY-N0322), Methyl-β-cyclodextrin (Mal-β-CD, MCE, HY-18593), AS1517499 (HY-100614), and CTPI-2 (HY-123986) were purchased from MedChemExpress (MCE, Shanghai, China) and were dissolved and stored according to the manufacturer's instructions. CHO was dissolved in ethanol as a 10 mM stock. Mal-β-CD and AS1517499 were prepared in DMSO as 50 mM stock solutions. To remove CHO from the medium, cells were pretreated with 5 mM Mal-β-CD for 2 h, followed by another 24-hour incubation in serum-free medium after which the conditioned supernatant was collected for co-culture. The SLC25A1 inhibitor CTPI-2 were dissolved in DMSO as 100 mM stocks. All compound stock solutions were stored at -20 °C.

### Data processing

Public expression profiles of endometriosis were obtained from the Gene Expression Omnibus (GEO) database (https://www.ncbi.nlm.nih.gov/geo/). Detailed information is presented in Table S2. Kyoto Encyclopedia of Genes and Genomes (KEGG) and Gene Set Enrichment Analysis (GSEA) were used to identify differential pathways via the “ClusterProfiler” R package.

### Cell transfection (gene knockdown and overexpression assays)

For SLC25A1 overexpression, the empty vector and the pLVX-IRES-neo-SLC25A1 overexpression plasmids were obtained from Jiangsu Gencefe Biotechnology Co., Ltd. (Jiangsu, China). For SLC25A1 knockdown, the pLKO.1-neo plasmids containing specific sequences targeting SLC25A1 (sequences: 5'- CCATCCGCTTCTTCGTCATGA-3' and 5'- ACACTCCTCTGGATGTGATTA-3') and the negative control plasmid were synthesized and purchased from Tsingke Biotech Co., Ltd. (Shanghai, China). Lentiviruses were generated by transfecting 293-T cells with the aforementioned plasmids and packaging plasmids (pMD2.G and psPAX2), and were then purified from the cell culture medium using a 0.45-μm strainer. Cells were seeded into 6-well plates and infected with the lentiviral suspension in the presence of 8 µg/mL polybrene (Beyotime, C0351, Shanghai, China) for 48 h, and then screened with 5 µg/mL G418 for 2 weeks (Beyotime, ST081, Shanghai, China).

### Quantitative real-time polymerase chain reaction (qRT-PCR)

Total RNA was extracted from tissues and cells by the TRIzol reagent (Takara, 9109, Tokyo, Japan) and reverse transcribed into cDNA using the PrimeScript™ RT reagent kit (ABclonal, RK20429, Wuhan, China). The qRT-PCR was performed using the SYBR Green qPCR Supermix kit (Abclonal, RK12106, Wuhan, China). The transcriptional levels of target genes were calculated using the 2^-ΔΔ^Ct method with β-actin as a comparative reference gene. The primer sequences are listed in Table S3.

### Western blotting (WB) assay

Total protein was extracted from cells and tissues using the RIPA lysis buffer (EpiZyme, WB3100, Shanghai, China) containing 1 % protease-phosphatase inhibitor (NCM Biotech, P002, Suzhou, China) and quantified using a bicinchoninic acid (BCA) Protein Assay Kit (ShareBio, SB-WB013, Shanghai, China). WB assay was performed according to previously described procedures 28, 29. Details of the antibodies are listed in Table S4.

### Immunohistochemistry (IHC) assay

IHC assay was performed as previously described 30. In brief, following deparaffinization and antigen retrieval, 4 % paraformaldehyde-fixed tissue samples were incubated overnight at 4 °C with anti-SLC25A1 antibody (1:200; Proteintech, 15235-1-AP, AB_2254794, Shanghai, China). Subsequently, sections were incubated with secondary antibody for 1 h at room temperature. Following the staining and counterstaining procedures, tissue sections were imaged and analyzed.

### RNA sequencing analysis

HESCs transfected with shSLC25A1 or treated with CTPI-2 (at ½ IC_50_ for 24 hours), and their corresponding control cells were subjected to RNA sequencing. Total RNA was extracted using the TRIzol reagent (Takara, 9109, Tokyo, Japan). Following RNA integrity assessment and RNA quantification using the Agilent 2100 Bioanalyzer (Agilent Technologies, Santa Clara, CA, USA) and the NanoDrop 2000 spectrophotometer (Thermo Scientific, USA), respectively, samples were dispatched to OE Biotech Co., Ltd. (Shanghai, China) for library construction with the VAHTS Universal V10 RNA-seq Library Prep Kit (Premixed Version) according to the manufacturer's instructions. Subsequently, the libraries were sequenced on the Illumina Novaseq 6000 platform to generate 150 bp paired-end reads. GSEA was performed to investigate the impact of SLC25A1 knockdown or inhibition.

### Quantification of cholesterol levels

The total cholesterol levels in tissues, cells and supernatants were detected using the Amplex Red Cholesterol and Cholesteryl Ester Assay Kit (Beyotime, S0211S, Shanghai, China). According to the manufacturer's instructions, cell supernatants from 1 × 10⁶ cells cultured for 48 h were collected, and 1 × 10^6^ cells or 50 mg tissues were lysed using BeyoLysis™ Buffer. Then all samples (including lysates and supernatants) were mixed with a cholesterol working solution and reacted at 37 ℃ for 30 min in the dark. Subsequently, the absorbance at 570 nm was measured using a multimode microplate reader (SpectraMax i3x, MOLECULAR DEVICES, USA) and normalized against a standard curve.

### Immunofluorescence assay

For BODIPY493/503 staining, cells were incubated with 2 µM BODIPY493/503 methyl bromide (MCE, HY-D1614, Shanghai, China) at 37 ℃ for 30 min in the dark and stained with Antifade Mounting Medium containing DAPI (Beyotime, P0131, Shanghai, China). For Filipin III staining, 4 % paraformaldehyde-fixed cells were incubated with 0.05 mg/ml Filipin III (MCE, HY-N6718, Shanghai, China) at room temperature for 2 h, and blocked with Antifade Mounting Medium containing PI (Beyotime, P0135, Shanghai, China). Images were captured via confocal microscope and analyzed by Image J software.

### Cell proliferation assays

For the CCK-8 assay, 2 × 10^3^ cells/well were seeded into 96-well plates and incubated with CCK-8 solution (CCK-8 reagent: medium=1:9) (NCM Biotech, C6005, Suzhou, China) for 2 h each day for five consecutive days. Absorbance was measured at 450 nm. For the colony formation assay, 1 × 10^3^ cells/well were seeded into 6-well plates and cultured for 14 days. Subsequently, the colonies were fixed with 4 % paraformaldehyde and stained with 1% crystal violet solution for 15 min. The colonies were photographed and then calculated using Image J.

### Cell invasion assays

For the Transwell assay, 4 × 10^4^ cells per well were seeded into the upper chamber of 24-well Transwell plates (Corning, 3422, NY, USA) in an FBS-free medium with or without Matrigel (BD, 356234, NY, USA) coating. After 24-48 h, the cells that migrated and invaded to the lower surface were stained with crystal violet and photographed under a microscope. For the wound-healing assay, near-confluent cells were scratched with a sterile tip and photographed at 0 and 12 h using a microscope. The acquired images were analyzed using the Image J software.

### Animal experiments

All animal experiments were approved by the Ethics Committee of the Tongji University. Six-week-old C57BL/6 female mice were purchased from the Shanghai JieSiJie Laboratory. The donor mice received 3 μg/mouse 17-β estrogen via intramuscular injection into the left hind limb three times per week. On day 7, the uteri of the donor mice were cut into debris and injected into the peritoneal cavity of the recipient mice. After 2 weeks, the mice were randomized and injected with CTPI-2 (50 mg/kg), or cholesterol (20 mg/kg) for an additional two weeks. CTPI-2 and CHO were formulated in a vehicle of 10% DMSO or 10% ethanol, respectively, along with 40% PEG300, 5% Tween-80, and 45% saline for intraperitoneal administration at a dose of 100 μL per 20 g mouse. Subsequently, the mice were sacrificed, and the ectopic lesions were harvested, weighted, and fixed for IHC assay.

### Statistical analysis

All experiments were conducted independently at least three times. The Student's t-test or one-way ANOVA were used to compare continuous variables. The results were analyzed and visualized using GraphPad Prism software V8.0, and presented as mean ± standard deviation (SD). *P* < 0.05 was considered statistically significant.

## Results

### Cholesterol accumulation promoted endometriosis progression in lesions

**Figure 1:**
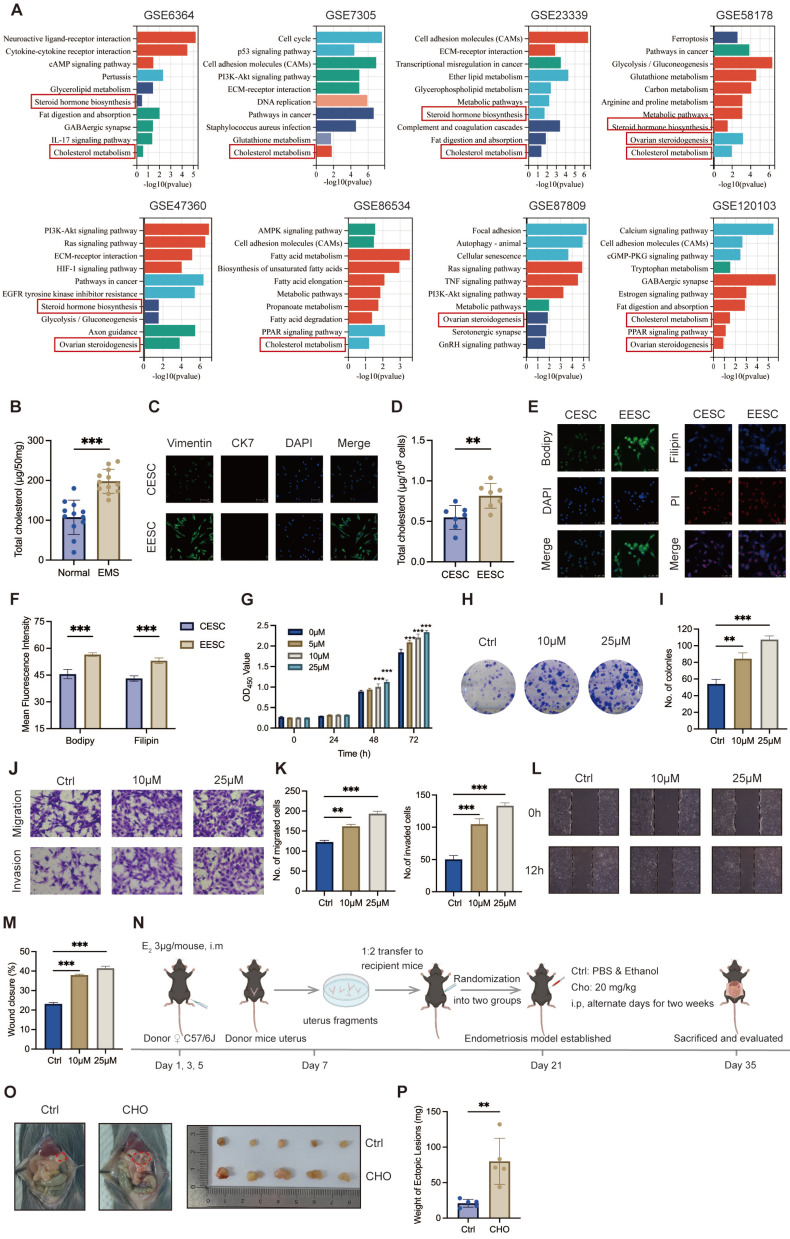
** Cholesterol accumulates in endometriotic lesions and promotes endometriosis progression. (A)** GSEA of eight endometriosis-related GEO datasets. **(B)** Cholesterol accumulated in endometriotic tissues (n=12, 50 mg per sample) compared with control endometrial tissues (n=12, 50 mg per sample). **(C)** Identification of isolated primary ESCs via immunofluorescence assay. Scale bar: 50 μm. **(D)** Intracellular cholesterol levels were significantly higher in EESCs (n=6) compared to those in CESCs (n=6). **(E)** BODIPY493/503 and Filipin III staining of primary ESCs by immunofluorescence assay. Scale bar: 50 μm. **(F)** The corresponding quantitative results of **(E)**. **(G)** The cell viability of EESCs treated with 0, 5, 10, and 25 μM cholesterol for 24 h, 48 h, and 72 h, respectively. **(H)** Cholesterol (10 μM and 25 μM, 48 h) treatment enhanced the proliferative capacity of EESCs by the colony formation assay. **(I)** Quantitative analysis of colony numbers from **(H)**. **(J-M)** Cholesterol (10 μM and 25 μM, 48 h) treatment promoted the migration and invasion abilities of EESCs by Transwell assay **(J, K)** and wound-healing assay **(L, M)**. **(N)** Diagram for constructing endometriosis mouse models. The intraperitoneal injection of free cholesterol was used to model a high-cholesterol peritoneal microenvironment, rather than to directly replicate EESCs-derived cholesterol secretion. **(O, P)** Representative images **(O)** and weight **(P)** of ectopic lesions. EMS, endometriosis; CESCs, control endometrial stromal cells; EESCs, ectopic endometrial stromal cells; CHO, cholesterol. ∗ *P* < 0.05, ∗∗ *P* < 0.01, ∗∗∗ *P* < 0.001.

To investigate essential regulation underlying endometriosis progression, we first examined the potential differences in crucial pathways between normal endometrial tissues and endometriotic lesions using GSEA on eight GEO datasets of endometriosis, and the results revealed that cholesterol metabolism was positively correlated with endometriosis (Figure 1A). Further analysis demonstrated that cholesterol significantly accumulated in endometriotic lesions compared with control endometrial tissues (Figure 1B). Primary ESCs, characterized by Vimentin positivity and Cytokeratin 7 negativity, were isolated from six control endometria and six of ectopic lesions (Figure 1C). Consistently, cellular cholesterol concentrations were substantially elevated in EESCs compared with CESCs (Figure 1D). Additionally, assessment of cholesterol content through Immunofluorescence assay revealed that EESCs had increased cholesterol content when compared to CESCs (Figure 1E, F). Thus, these results demonstrated that cholesterol accumulates in the ectopic tissues and EESCs.

Next, we explored the biological functions of cholesterol in endometriosis. To determine the impact of cholesterol on the growth of EESCs, CCK-8 and colony formation assays were performed. CCK-8 assay revealed that cholesterol significantly promoted EESCs growth in a dose- and time-dependent manner (Figure 1G). Similarly, colony formation assay also demonstrated that cholesterol significantly augmented the proliferative capacity of EESCs (Figure 1H, I). Moreover, cholesterol treatment led to a significant increase in the migration and invasion capacities of EESCs (Figure 1J-M). Additionally, allograft endometriosis models were established and randomized into cholesterol and control groups (Figure 1N). The cholesterol group exhibited a substantial increase in the weight of endometriotic lesions, indicating that elevated cholesterol concentrations in the mouse peritoneal cavity may promote endometriosis development (Figure 1O, P). Taken together, these data indicated that cholesterol facilitates endometriosis progression both *in vitro* and *in vivo*.

### EESCs-derived cholesterol mediated M2 macrophage polarization in the endometriotic milieu

**Figure 2:**
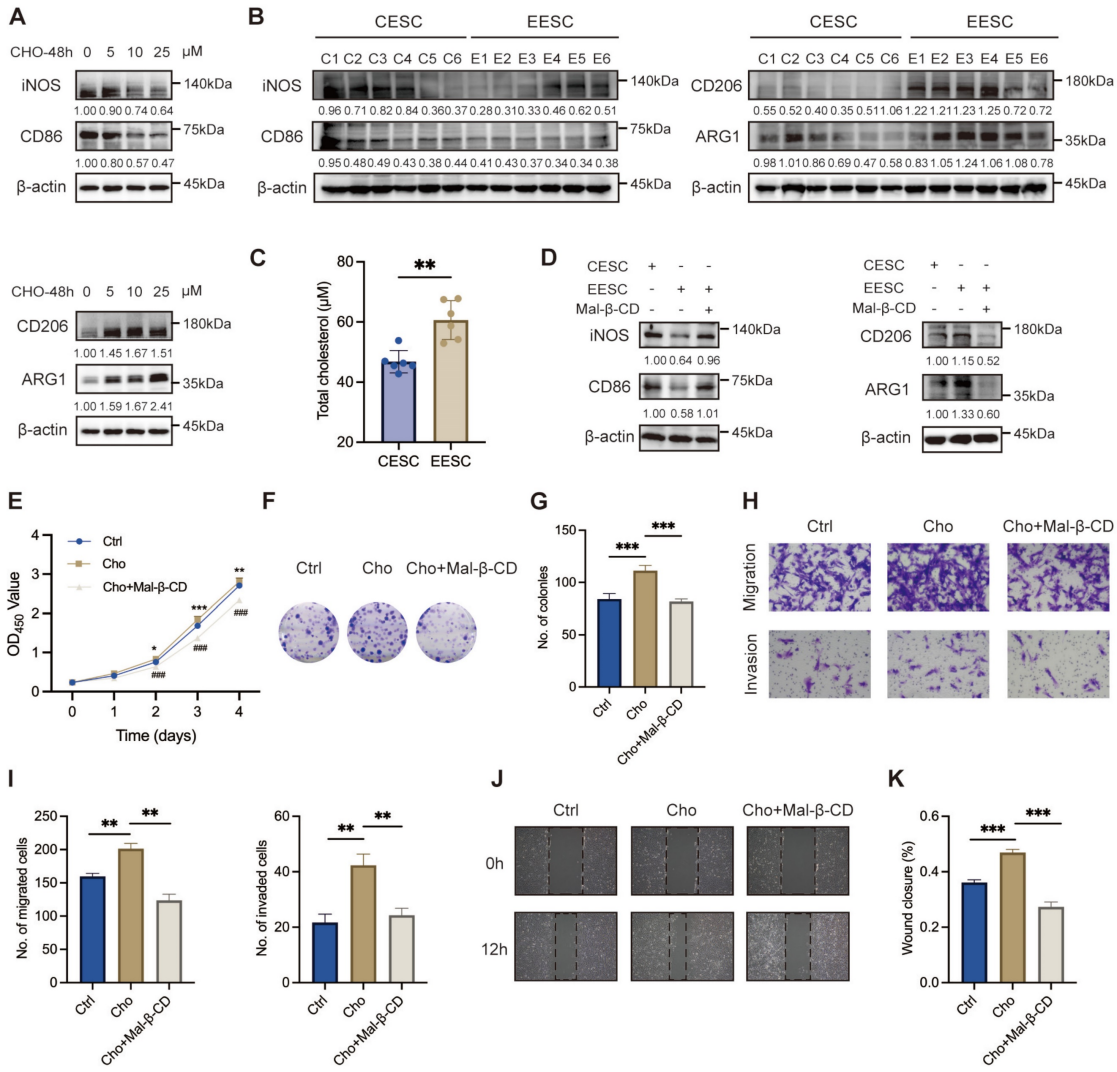
** Cholesterol boosts M2 phenotype polarization in endometriosis. (A)** The protein levels of M1 markers (iNOS and CD86) and M2 markers (CD206 and ARG1) treated with cholesterol (0, 5, 10, and 25 μM) for 48 h. **(B)** The protein levels of M1 markers (iNOS and CD86) and M2 markers (CD206 and ARG1) in macrophages co-cultured with CESCs and EESCs supernatants. **(C)** Cholesterol concentration in the culture supernatants of CESCs (n=6) and EESCs (n=6). **(D)** The protein levels of M1 markers (iNOS and CD86) and M2 markers (CD206 and ARG1) in macrophages co-cultured with CESCs supernatants or EESCs supernatants (with or without Mal-β-CD, 5 mM for 2 h). **(E-H)** CCK-8 assay **(E)**, colony formation assay **(F, G)**, Transwell assay **(H, I)**, and wound-healing assay **(J, K)** were performed to assess the proliferation, migration, and invasion of EESCs in control and cholesterol-induced (with or without Mal-β-CD, 5 mM for 2 h) macrophages conditioned medium. ∗ *P* < 0.05, ∗∗ *P* < 0.01, ∗∗∗ *P* < 0.001.

Macrophages contribute to the initiation and exacerbates the progression of endometriotic lesions. Cholesterol has been reported to play an essential role in macrophage polarization 31. To compare the mRNA expression levels between M1 and M2 markers after cholesterol treatment, we performed qRT-PCR on M0 macrophages, which were derived from THP-1 cells and exposed to 0, 5, 10, and 25 μM cholesterol concentrations for 48 h. As depicted in Figure S1A-B, cholesterol exposure induced a significant upregulation of most M2 signature genes and downregulation of M1 signature genes. We noticed that a low concentration of cholesterol (5 μM) significantly induced the transcription of proinflammatory factors (TNF-α, IL-1α, IL-1β, IL-6, and IL-10) in macrophages. By contrast, elevating the cholesterol concentration to 10 μM and 25 μM led to an opposite regulatory effect. WB demonstrated that cholesterol-induced decreases in the expressions of M1 markers (iNOS and CD86) and increases in that of M2 markers (CD206 and ARG1), in a dose-dependent manner (Figure 2A).

To further investigate the relationship between cholesterol and macrophage polarization in endometriosis, we detected the expression levels of M1 and M2 markers following the co-culturing of primary ESCs supernatant with M0 macrophages. Compared with the CESCs supernatants, the EESCs supernatants significantly elevated the expression of M2 markers while reduced the expression of M1 markers, implying that the EESCs supernatants induced M2 macrophage polarization (Figure S1C-D, Figure 2B). Following the collection of the conditioned media, we observed that the cholesterol concentration in EESCs culture supernatants were significantly higher than those in CESCs (Figure 2C). Therefore, we speculated that cholesterol present in the supernatants of EESCs drives macrophages toward M2 polarization. To validate this hypothesis, EESCs were pretreated with 5 mM Mal-β-CD for 2 h to deplete the cholesterol in the medium, then the supernatant was collected for co-culture with M0 macrophages. Similar results were obtained when comparisons were made between the CESCs supernatant group and the EESCs with Mal-β-CD treatment group. Specifically, Mal-β-CD partially inhibited the promotion of M2 polarization by the EESCs supernatants (Figure S1E-F, Figure 2D). These results indicate that cholesterol may drive macrophage polarization towards an M2 phenotype within the endometriotic microenvironment.

### Cholesterol-induced M2 macrophage polarization enhanced endometriosis progression

We further elucidate the biological impact of cholesterol-induced M2 macrophages on endometriosis progression. To mimic the ectopic immune microenvironment, we established a co-culture model integrating EESCs and macrophages under cholesterol treatment (with or without Mal-β-CD). Conditioned medium obtained from cholesterol-activated macrophages induced a significantly increase in the proliferation, migration, and invasion of EESCs than that from control macrophages. Notably, Mal-β-CD treatment partly reversed these effects that were related to the enhancement of endometriosis progression (Figure 2E-K). Therefore, cholesterol production by EESCs and M2 polarization of macrophages seem to exhibit a mutually reinforcing relationship that results in a vicious cycle in the endometriotic milieu, accelerating the progression of endometriosis.

### Cholesterol facilitated M2 macrophage polarization via the STAT6/PPARγ pathway

**Figure 3:**
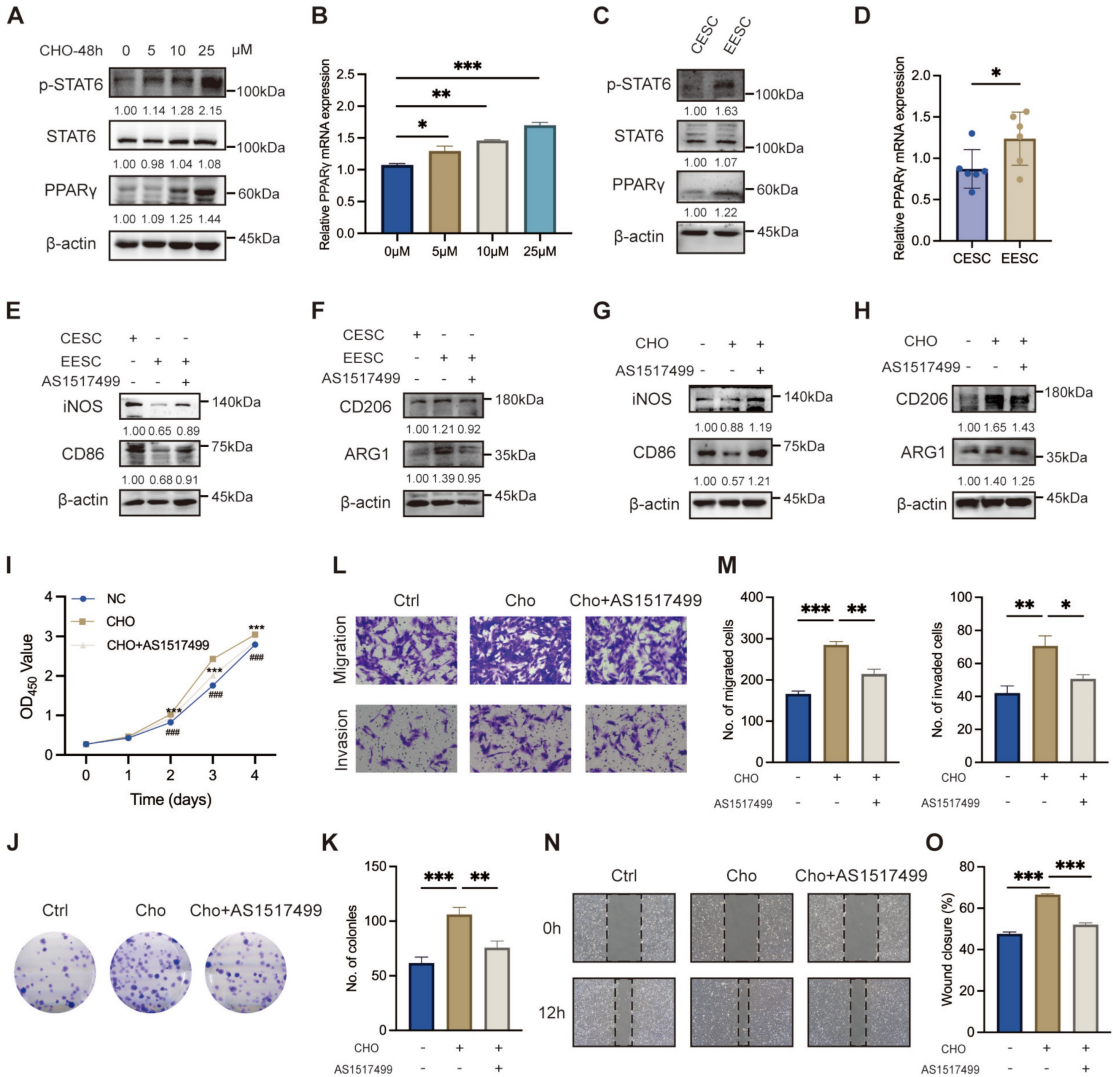
** Cholesterol facilitates M2 macrophage polarization via the STAT6/PPARγ pathway. (A)** The protein levels of p-STAT6, STAT6, and PPARγ following treatment with various concentrations of cholesterol (0, 5, 10, and 25 μM) for 48 h. **(B)** The mRNA levels of PPARγ after treatment with various concentrations of cholesterol (0, 5, 10, and 25 μM) for 48 h. **(C)** The protein levels of p-STAT6, STAT6, and PPARγ after co-culture with supernatants from CESCs or EESCs. **(D)** The mRNA level of PPARγ after co-culture with supernatants from CESCs or EESCs. **(E, F)** The protein levels of M1 and M2 markers in macrophages co-cultured with supernatants from CESCs, or EESCs (with or without AS1517499 pretreatment). **(G, H)** The protein levels of M1 and M2 markers in cholesterol treated macrophages following pretreatment with 1 μM AS1517499 for 2 h. **(I-O)** CCK-8 assay **(I)**, colony formation assay **(J, K)**, Transwell assay **(L, M)**, and wound-healing assay **(N, O)** were conducted to evaluate the proliferation, migration, and invasion abilities of EESCs co-cultured with control and cholesterol-treated (with or without AS1517499 pretreatment) conditioned supernatants from THP-1-derived macrophages. ∗ *P* < 0.05, ∗∗ *P* < 0.01, ∗∗∗ *P* < 0.001.

Previous studies have demonstrated that STAT6 effectively facilitates macrophage differentiation into the M2 phenotype 32-34. As depicted in Figure 3A, the protein levels of p-STAT6 and PPARγ in macrophage exhibited a significant upregulation along with the increasing cholesterol concentrations. Simultaneously, PPARγ mRNA levels were elevated (Figure 3B). Moreover, compared with the supernatant of CESC, the supernatant of EESC significantly activated the expression of pSTAT6 and PPARγ (Figure 3C, D). Therefore, we hypothesized that accumulated cholesterol promoted STAT6 phosphorylation and activated PPARγ transcription and expression. To validate our hypothesis, macrophages were pre-incubated with AS1517499 (a specific STAT6 inhibitor, 1 μM) for 2 h. As expected, AS1517499 effectively inhibited cholesterol-mediated phosphorylation of STAT6 and subsequent upregulation of PPARγ expression (Figure S2A-B). Consistently, the activation of the STAT6/PPARγ pathway induced by EESC supernatant was also blocked by AS1517499 (Figure S2C-D). Moreover, AS1517499 treatment led to a significant suppression of both EESC supernatant-induced (Figure S2E-F, Figure 3E-F) and cholesterol-driven M2 polarization (Figure S2G-H, Figure 3G-H). Additionally, the findings obtained in THP-1 cells were further validated in RAW264.7 cells (Figure S2I-J). Meanwhile, THP-1-derived macrophage culture supernatants following cholesterol treatment (with or without AS1517499 preincubation) were collected and co-cultured with EESCs. We found that AS1517499 partially reversed the promotion of EESC proliferation, migration, and invasion induced by the supernatant of cholesterol-stimulated macrophages (Figure 3I-O). Collectively, these findings demonstrated that cholesterol facilitates M2 macrophage polarization, and EESCs proliferation and invasion by activating STAT6 and its downstream effector, PPARγ.

### SLC25A1 contributed to cholesterol accumulation in ESCs

**Figure 4:**
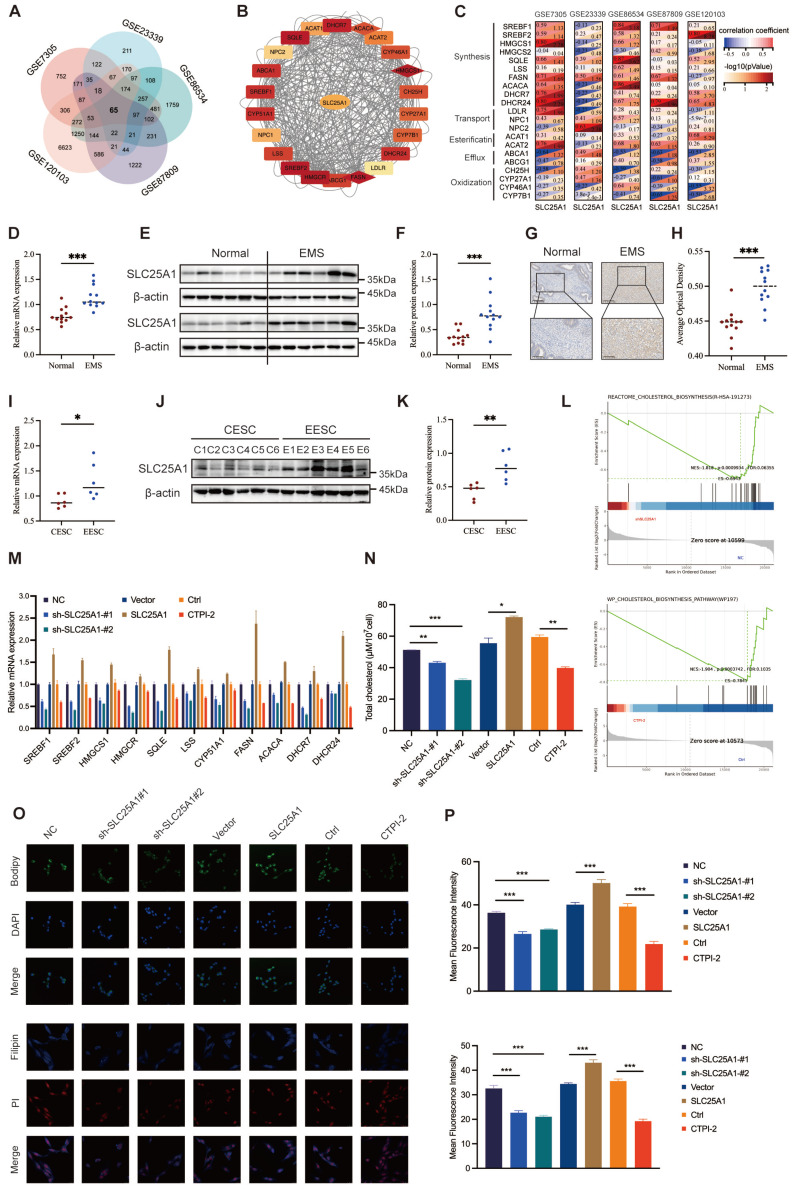
** SLC25A1 contributes to cholesterol metabolism in ESCs. (A)** Venn diagram of differentially expressed genes of five endometriosis datasets (GSE7305, GSE120103, GSE23339, GSE6364, and GSE87809). **(B, C)** The protein-protein interaction network and correction analysis of SLC25A1 and cholesterol metabolism-related genes. **(D-H)** The mRNA and protein levels of SLC25A1 in endometriotic lesions (n=12) and control endometrial tissues (n=12) were measured using qRT-PCR **(D)**, WB **(E, F)**, and IHC **(G, H)**. Scale bar: 100 μm **(I, K)** The mRNA **(I)** and protein **(J, K)** levels of SLC25A1 in CESCs (n=6) and EESCs (n=6). **(L)** GSEA in the control group and the shSLC25A1 or the CTPI-2 treatment group. **(M)** The mRNA expression of cholesterol synthesis-related genes in the SLC25A1 knockdown, SLC25A1 overexpression, CTPI-2 treatment, and their respective control groups. **(N)** The cholesterol content in the supernatants from HESCs (shSLC25A1, OE-SLC25A1, CTPI-2 treatment, and their respective control groups). For the CTPI-2 treatment group, to rule out the direct effect of CTPI-2 in HESC supernatants on THP-1 cells, we treated HESCs with ½ IC₅₀ CTPI-2 for 24 h, then replaced the medium with complete medium, and continued culturing the HESCs for another 24 h. **(O)** Immunofluorescence of BODIPY493/503 and Filipin III staining on HESCs (shSLC25A1, OE-SLC25A1, CTPI-2 treatment, and their respective control groups). Scale bar: 50 μm. **(P)** The corresponding quantitative results of **(O)**. ∗ *P* < 0.05, ∗∗ *P* < 0.01, ∗∗∗ *P* < 0.001.

To further uncover the underlying mechanisms of dysregulated cholesterol in endometriosis, we analyzed five transcriptome profiles of endometriosis from the GEO database and obtained 65 candidate genes that were differentially expressed across all datasets (Figure 4A). Subsequently, we calculated the correlations between the candidate genes and the genes related to cholesterol metabolism (including synthesis, transport, esterification, efflux, and oxidization), among which SLC25A1 showed a remarkable correlation (Figure 4B-C). To assess SLC25A1 expression in clinical samples, qRT-PCR (Figure 4D), WB (Figure 4E, F), and IHC (Figure 4G, H) were performed on endometriotic (n = 12) and control tissues (n = 12). The results demonstrated that the mRNA and protein levels of SLC25A1 were significantly upregulated in ectopic endometria (Figure 4D-H). Consistent with the result in clinical tissues, SLC25A1 mRNA and protein levels in EESCs were also higher than those in CESCs (Figure 4I-K).

As depicted in Figure S3A-B, we successfully constructed stable SLC25A1 knockdown and overexpression cell lines in HESCs, and verified their efficiency using qRT-PCR and WB. Meanwhile, we measured the half-maximal inhibitory concentration of CTPI-2 (IC_50_, 21.24 μM), an SLC25A1 competitive inhibitor, on HESCs, and found it had no impact on SLC25A1 expression at varying times and concentrations (Figure S3C-D). To further elucidate the function of SLC25A1 in cholesterol metabolism, we conducted GSEA in the control group and in the shSLC25A1 or the CTPI-2 treatment groups. SLC25A1 was considerably involved in cholesterol metabolism, and its downregulation significantly suppressed this process (Figure 4L). Furthermore, transcriptomic sequencing data revealed that the mRNA levels of most genes associated with cholesterol biosynthesis were significantly decreased in the SLC25A1 knockdown and CTPI-2 treatment groups compared to those in the control groups (Figure S3E). The results of qRT-PCR assays on ESCs further confirmed this finding (Figure 4M). Additionally, we found that SLC25A1 knockdown or CTPI-2 treatment significantly reduced the triglycerides and cholesterol contents of supernatants from HESCs, whereas SLC25A1 overexpression had the opposite effect (Figure S3F and Figure 4N). Meanwhile, BODIPY493/503 and Filipin III staining revealed that the intracellular contents of triglycerides and cholesterol in HESCs were consistent with the above results (Figure 4O, P). These findings indicated that SLC25A1 serves as a crucial driver in upregulating cholesterol metabolism in endometriosis.

### SLC25A1 promoted endometriosis progression *in vitro* and *in vivo*

**Figure 5:**
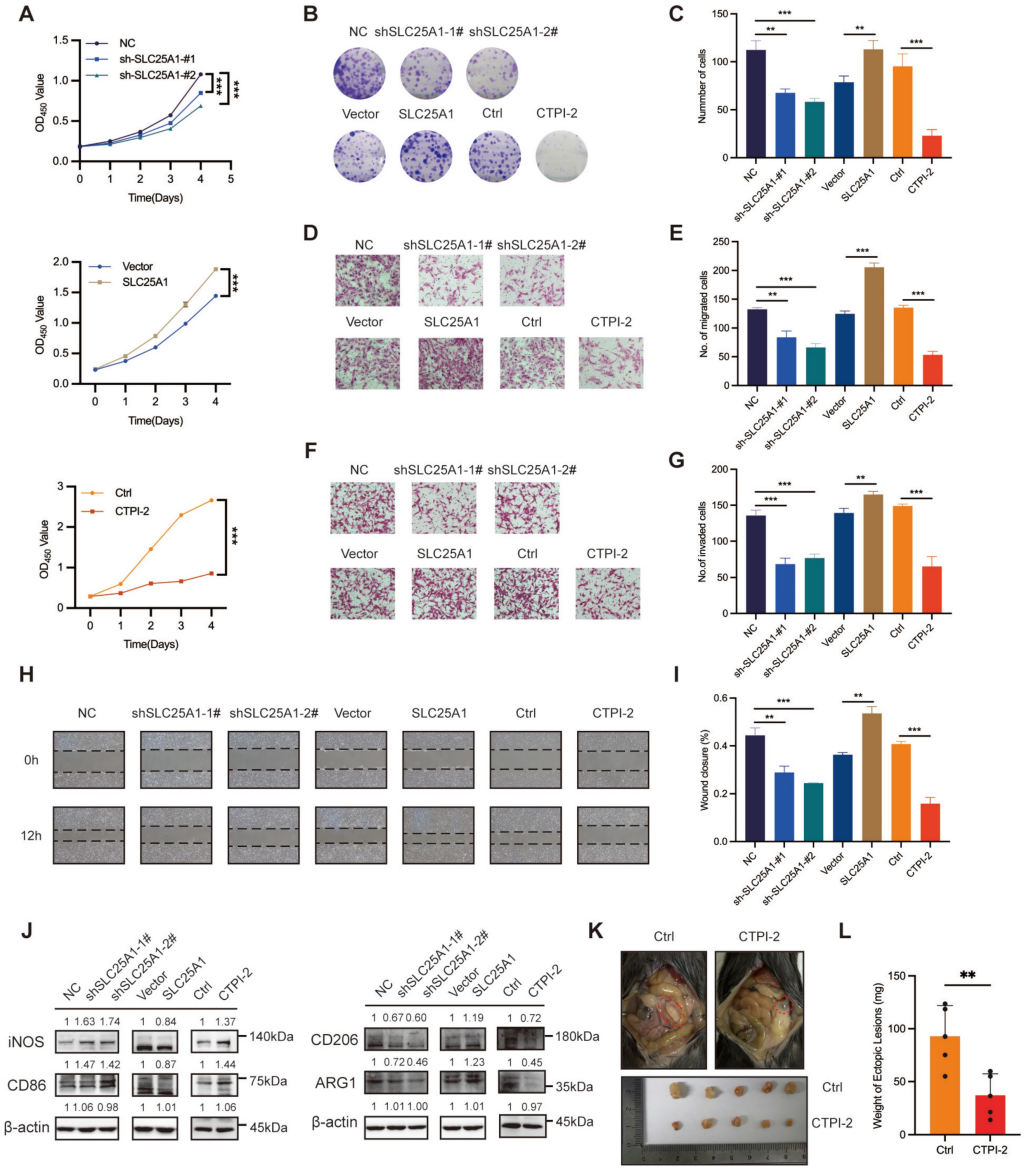
** SLC25A1 promotes endometriosis progression* in vitro* and *in vivo.* (A, B)** CCK-8 assay **(A)** and colony formation assay **(B, C)** were performed to detect the effect of SLC25A1 on the proliferation ability of HESCs. **(D-I)** Transwell assay **(D-G)** and wound-healing assay **(H, I)** were conducted to evaluate the impact of SLC25A1 on HESCs migration and invasion. **(J)** The protein levels of iNOS, CD86, CD206, and ARG1 in THP-1-derived macrophages co-cultured with supernatants from HESCs with SLC25A1 knockdown, SLC25A1 overexpression, or inhibition (CTPI-2 treatment), as well as their respective control groups. For the SLC25A1 inhibition group, to rule out the direct effect of CTPI-2 in HESC supernatants on THP-1 cells, we treated HESCs with ½ IC₅₀ CTPI-2 for 24 h, then replaced the medium with complete medium, and continued culturing the HESCs for another 24 h. **(K, L)** Representative images **(K)** and weights **(L)** of ectopic lesions of the control and CTPI-2 groups of the mouse endometriosis model. ∗ *P* < 0.05, ∗∗ *P* < 0.01, ∗∗∗ *P* < 0.001.

We investigated the biological effects of SLC25A1 in the context of endometriosis. SLC25A1 knockdown and CTPI-2 treatment (½ IC_50_, 24 h) significantly reduced the proliferation, migration and invasion of HESCs. Following SLC25A1 overexpression, HESCs exhibited remarkable increases in the ability of proliferation, migration and invasion (Figure 5A-I). Next, we assessed the differential effects of the cell supernatants obtained from HESCs subjected to stable SLC25A1 knockdown, overexpression, or CTPI-2 treatment on THP-1-derived macrophage polarization. As anticipated, in the co-culture system, SLC25A1 overexpression significantly suppressed the expression of M1 markers (especially iNOS and CD86) and enhanced the expression of M2 markers (particularly CD206 and ARG1) (Figure S4A-B). Conversely, SLC25A1 knockdown and inhibition led to the opposite results (Figure 5J). Furthermore, as shown in Figure S4C, we evaluated SLC25A1 function in an animal model of endometriosis. As expected, compared with mice in the control group, those in the CTPI-2 treatment group exhibited lighter ectopic lesions (Figure 5K and L). Taken together, these results indicate that SLC25A1 actively participates in cholesterol metabolism and promotes various endometriotic processes, including proliferation, migration, invasion, and M2 macrophage polarization.

### SLC25A1 accelerated endometriosis progression via regulating cholesterol metabolism

**Figure 6:**
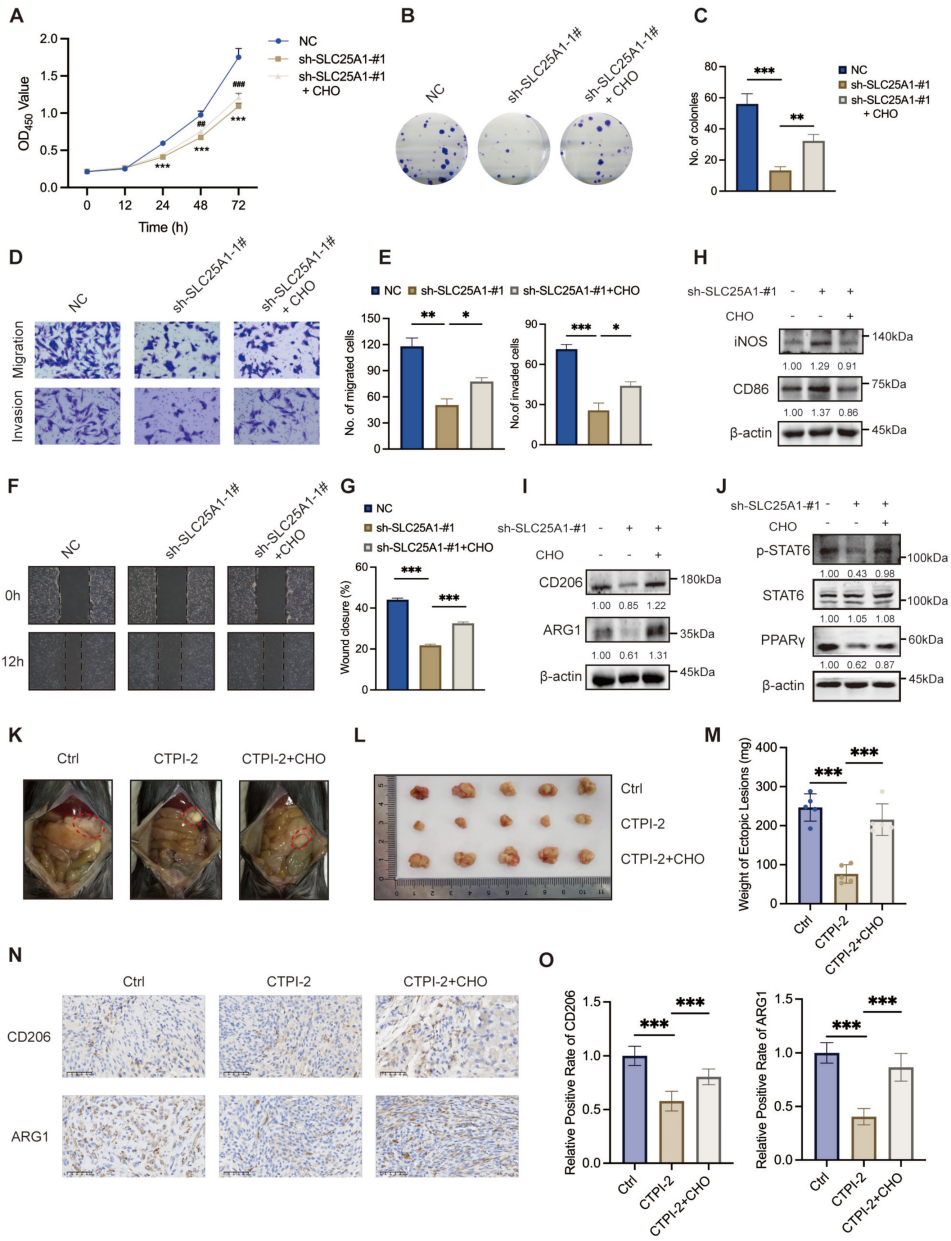
** SLC25A1 accelerates the endometriosis progression via cholesterol metabolism regulation. (A-C)** The addition of cholesterol (10 μM, 48 h) was able to partially reverse the anti-proliferative effect induced by SLC25A1 knockdown. **(D-G)** Transwell assay **(D, E)** and wound-healing assay **(F, G)** were conducted on HESCs with SLC25A1 knockdown, with or without cholesterol supplementation (10 μM, 48 h). **(H, I)** The protein levels of iNOS, CD86, CD206, and ARG1 in THP-1-derived macrophages co-cultured with supernatants from HESCs with SLC25A1 knockdown, with or without cholesterol supplementation. **(J)** The protein levels of p-STAT6, STAT6 and PPARγ in THP-1-derived macrophages co-cultured with supernatants from SLC25A1-knockdown HESCs (with or without cholesterol supplementation). **(K-M)** Representative images **(K, L)** and weights **(M)** of ectopic lesions. **(N)** IHC analysis of CD206 and ARG1 in lesional tissues. Scale bar: 50 μm. **(O)** The corresponding quantitative results of **(N)**. ∗ *P* < 0.05, ∗∗ *P* < 0.01, ∗∗∗ *P* < 0.001.

To verify whether SLC25A1 promoted the progression of endometriosis by upregulating cholesterol metabolism, we conducted rescue experiments by supplementing the shSLC25A1 group with cholesterol. CCK-8 and colony formation assays demonstrated that cholesterol supplementation partially reversed the antiproliferative effect induced by SLC25A1 knockdown (Figure 6A-C). Transwell and wound-healing assays yielded consistent results (Figure 6D-G). Additionally, using a co-culture model, we discovered that exogenous cholesterol was able to reverse the decline in M2 macrophage polarization induced by SLC25A1 knockdown (Figure S5A, Figure 6H-I). Moreover, supplementing the conditioned medium from SLC25A1-knockdown ESCs with exogenous cholesterol partially restored the activation of the STAT6-PPARγ pathway in macrophages (Figure 6J). *In vivo* experiment demonstrated that CTPI-2 treatment-induced reduction in the weight of endometriotic lesions was effectively reversed by cholesterol supplementation (Figure 6K-M). Furthermore, IHC analysis showed that CTPI-2 markedly reduced M2 macrophage infiltration (as indicated by CD206 and ARG1), whereas this effect was reversed by the simultaneous addition of cholesterol (Figure 6N, O). Overall, these results strongly suggested that SLC25A1 drives M2 macrophage polarization and endometriosis progression, with cholesterol metabolism being a key regulator of this process.

## Discussion

Cholesterol is a crucial cell membrane component that is indispensable for maintaining membrane integrity, mediating cell signaling, and participating in hormone synthesis 13. Research indicates that the homeostasis of cholesterol metabolism considerably impacts the malignant biological activities of cancer cells, including proliferation, migration, and invasion 35, 36. Additionally, cholesterol is intricately associated with tumor-infiltrating immune cells, which drive the induction and resolution of the inflammatory response 29, 37. Notably, aberrant cholesterol accumulation and metabolic dysregulation have also been implicated in chronic inflammatory conditions, including cardiovascular and neurodegenerative diseases 38.

Our study indicated that cholesterol accumulation in ectopic lesions and EESCs significantly promoted the proliferation, migration, and invasion of EESCs, ultimately promoting the progression of endometriosis. EESCs with a complete aromatase conversion system convert abundant local cholesterol into estrogen, thereby facilitating the sustained growth and invasion of endometriotic lesions 39. As endometriosis progresses, the excessive accumulation of cholesterol within ectopic lesions could disrupt the immune microenvironment and accelerate endometriosis deterioration 40. Macrophages are essential constituents of the endometriotic microenvironment 21. Specifically, M2 macrophages hold a notably prominent position because of their substantial roles in tissue repair and immunosuppressive actions 41. Peritoneal fluid of endometriosis patients contains significantly more M2 macrophages than that of patients without endometriosis 42. Our study observed a biphasic macrophage response to cholesterol, with a low-dose cholesterol (5 μM) inducing pro-inflammatory activation (increased M1 macrophage polarization and cytokines) and high doses (10-25 μM) shifting the balance toward M2 polarization. We found that in the microenvironment of endometriosis, cholesterol mediated the polarization of macrophages into the M2 phenotype to inhibit immune cell activation and decrease immune clearance efficacy via the secretion of various cytokines (including IL-10 and TGF-β), facilitating the survival and progression of endometriotic lesions. More importantly, the cholesterol-induced polarization of M2 macrophages further accelerated the deteriorating course of endometriosis. This occurred as M2 macrophages promoted the proliferation and invasion of EESCs via intercellular secretory signaling, inflammatory mediators, and growth factors. In turn, EESCs-derived chemokines promoted macrophage polarization in ectopic lesions, where the infiltrated macrophages were exposed to cholesterol. This positive feedback loop continuously amplifies the promoting effects of cholesterol accumulation and M2 macrophages on endometriosis, exacerbating the inflammatory response and tissue damage, propelling the continuous progression and exacerbation of endometriosis, and consequently posing a severe threat to the health of patients. Therefore, elucidating the role of cholesterol metabolism in endometriosis and developing innovative cholesterol-targeting strategies may provide new insights into endometriosis management.

SLC25A1, a key member of the mitochondrial carrier protein family, assumes a pivotal position in maintaining cellular metabolic homeostasis, and its dysfunction is closely associated with metabolic disorders and tumor development 43-45. Previous studies have shown that SLC25A1 enhances citrate transport to support the de novo synthesis of cholesterol 46, 47. In our study, SLC25A1 was highly upregulated in ectopic lesions, closely linked to cholesterol metabolism-related genes, and significantly regulated intracellular and extracellular cholesterol content. In terms of cellular function, SLC25A1 significantly promoted proliferation, migration and invasion of HESCs, and drove macrophage polarization towards the M2 phenotype via the regulation of cholesterol metabolism, creating a favorable immune microenvironment for endometriosis progression. Meanwhile, *in vitro* and *in vivo* experiments demonstrated that cholesterol supplementation reversed the cellular and immune microenvironment alterations caused by SLC25A1 knockdown, further confirming the critical role of SLC25A1 in the endometriosis progression mediated by cholesterol metabolism. Our results suggest that SLC25A1 is a key target in the regulation of cholesterol metabolism and endometriosis progression. However, the sample size of our study is relatively small, and further validation with large samples is required. Furthermore, the exact mechanisms underlying SLC25A1-mediated regulation of cholesterol metabolism remain to be elucidated. Additionally, the partial rescue effects of exogenous cholesterol supplementation indicate that while cholesterol metabolism is a key SLC25A1-regulated downstream pathway in endometriosis, it may not be the sole mediator of the biological functions of SLC25A1. Given that SLC25A1 also has complex functions such as regulating fatty acid synthesis and maintaining redox homeostasis, this warrants further exploration.

In the complex microenvironment of endometriosis, except for exerting a direct promoting impact on EESCs, cholesterol drives the polarization of macrophages towards the M2 phenotype through the STAT6/PPARγ signaling pathway. We hypothesize that cholesterol accumulated in ectopic lesions phosphorylates and subsequently activates STAT6, which translocates to the nucleus to initiate PPARγ transcription and translation in macrophages. This process further regulates the expression of genes related to macrophage polarization and the release of inflammatory factors to promote M2 macrophage polarization, thereby facilitating endometriosis progression 48-50. Our findings suggest that obstructing the association between cholesterol and macrophage activity may serve as a promising strategy for remodeling the immune microenvironment and decelerating the progression of endometriosis.

## Conclusion

In summary, our results demonstrated that cholesterol accumulation in endometriotic lesions directly promotes ESCs proliferation and invasion, and stimulates M2 macrophage polarization via the STAT6/PPARγ signaling pathway, creating a microenvironment conducive to endometriosis progression. SLC25A1 upregulates cholesterol metabolism in ESCs and directly exacerbates endometriosis deterioration. Consequently, disrupting the pernicious cycle involving SLC25A1-mediated cholesterol accumulation in ESCs and cholesterol-induced M2 macrophage polarization may lead to new breakthroughs in endometriosis therapy.

## Supplementary Material

Supplementary figures and tables.

## Abbreviations

EESCs: ectopic endometrial stromal cells
SLC25A1: solute carrier family 25 member 1
ESCs: endometrial stromal cells
CESCs: control endometrial stromal cells
CHO: cholesterol
KEGG: Kyoto Encyclopedia of Genes and Genomes
GSEA: Gene Set Enrichment Analysis
GEO: Gene Expression Omnibus
