# Supplementary material for: SLC25A1-Mediated Cholesterol Accumulation Promotes Endometriosis Progression by Enhancing Endometrial Stromal Cell Proliferation, Invasion, and M2 Macrophage Polarization
Source: Int J Biol Sci. 2026 Jan 1;22(1):466–80. doi: 10.7150/ijbs.117146 (PMC12681928; doi:10.7150/ijbs.117146)
Supplement: Supplementary file 1 — Supplementary figures and tables. [file ijbsv22p0466s1.pdf]

Supplementary materials

Supplementary Tables

Table S1. Detailed information of clinical samples in this study

Group	ID	Diagnosis	Age (years)	Dysmenorrhea	Stage	Utilization
Normal	NC-1	Tubal infertility	28	No	/	qRT-PCR, WB, IHC, CHO content detection
	NC-2		25	Yes	/	qRT-PCR, WB, IHC, CHO content detection
	NC-3		32	No	/	ESC isolation
	NC-4		27	No	/	qRT-PCR, WB, IHC, CHO content detection
	NC-5		35	Yes	/	qRT-PCR, WB, IHC, CHO content detection
	NC-6		33	Yes	/	qRT-PCR, WB, IHC, CHO content detection
	NC-7	Uterine septum	27	No	/	ESC isolation
	NC-8		31	No	/	qRT-PCR, WB, IHC, CHO content detection
	NC-9		30	Yes	/	qRT-PCR, WB, IHC, CHO content detection
	NC-10		26	Yes	/	qRT-PCR, WB, IHC, CHO content detection
	NC-11	Endometrial polyps	31	No	/	ESC isolation
	NC-12		26	Yes	/	qRT-PCR, WB, IHC, CHO content detection
	NC-13		42	Yes	/	qRT-PCR, WB, IHC, CHO content detection
	NC-14		44	No	/	ESC isolation
	NC-15		39	Yes	/	ESC isolation
	NC-16		37	No	/	qRT-PCR, WB, IHC, CHO content detection
	NC-17		44	Yes	/	qRT-PCR, WB, IHC
	NC-18		38	Yes	/	ESCs isolation
EMS	EC-1	Ovarian endometriosis	26	No	IV	qRT-PCR, WB, IHC, CHO content detection
	EC-2		27	No	III	qRT-PCR, WB, IHC, CHO content detection
	EC-3		29	Yes	IV	ESC isolation
	EC-4		29	Yes	IV	ESC isolation
	EC-5		30	No	III	qRT-PCR, WB, IHC, CHO content detection
	EC-6		30	Yes	III	qRT-PCR, WB, IHC,

				CHO content detection
EC-7	31	No	IV	ESC isolation
EC-8	32	Yes	IV	qRT-PCR, WB, IHC, CHO content detection
EC-9	33	Yes	III	qRT-PCR, WB, IHC, CHO content detection
EC-10	33	Yes	IV	qRT-PCR, WB, IHC
EC-11	35	No	IV	ESC isolation
EC-12	35	Yes	III	qRT-PCR, WB, IHC, CHO content detection
EC-13	37	Yes	III	qRT-PCR, WB, IHC, CHO content detection
EC-14	39	Yes	III	qRT-PCR, WB, IHC, CHO content detection
EC-15	40	No	IV	ESC isolation
EC-16	41	Yes	IV	qRT-PCR, WB, IHC, CHO content detection
EC-17	43	No	IV	qRT-PCR, WB, IHC, CHO content detection
EC-18	44	No	IV	qRT-PCR, WB, IHC, CHO content detection

EMS, Endometriosis; NC, normal endometria; EC, ectopic endometria; ESC, endometrial stromal cell; qRT-PCR, quantitative real-time polymerase chain reaction; WB, Western blotting; IHC, Immunohistochemistry; CHO, cholesterol.

Table S2. Detailed information of GEO datasets

GSE	Platform	Type	Samples
GSE6364	GPL570	Tissue	Normal (16) and EMS (21)
GSE7305	GPL570	Tissue	Normal (10) and EMS (10)
GSE23339	GPL6102	Tissue	Normal (9) and EMS (10)
GSE58178	GPL6947	ESCs	Normal (6) and EMS (6)
GSE47360	GPL6244	ESCs	Normal (3) and EMS (3)
GSE86534	GPL20115	Tissue	Normal (4) and EMS (4)
GSE87809	GPL11154	ESCs	Normal (5) and EMS (4)
GSE120103	GPL6480	Tissue	Normal (18) and EMS (18)

EMS, Endometriosis; ESCs, endometrial stromal cells.

Table S3. The primer sequences used for qRT-PCR

Gene	Forward-Sequence (5'-3')	Reverse-Sequence (5'-3')
β-actin	CACTCTTCCAGCCTTCCTTC	GTACAGGTCTTTGCGGATGT
SLC25A1	CCCCATGGAGACCATCAAG	CCTGGTACGTCCCCTTCAG

SREBF2	ACAACCCATAATATCATTGAGAAACG	TTGTGCATCTTGGCGTCTGT
HMGCS	AAAGTACCAAGACTCCCTGCCACA	ATCCCATTCCTCCAACTGTTCCCA
LSS	CATCAACATGCTTGTGCGCTGGTA	ATTTTCATGCCGTCAAGGCCCATC
SQLE	CTATGGCAGAGCCCAATGCAAAGT	ACAACAGTCAGTGGAGCATGGAGT
CYP51A1	GAGTGATGCTGCTGCACTGCTTTT	ACATCGTATGCAACTCCCTTCCCA
HMGCR	GCCTGGCTCGAAACATCTGAA	CTGACCTGGACTGGAAACGGATA
DHCR7	CAGCGCCAGAGACTGCAAA	TGAAGAACAGCTTGAAGTCAAACC
SREBF1	CGGAACCATCTTGGCAACAGT	CGCTTCTCAATGGCGTTGT
FASN	CCGAGACACTCGTGGGCTA	CTTCAGCAGGACATTGATGCC
ACACA	ATGTCTGGCTTGCACCTAGTA	CCCCAAAGCGAGTAACAAATTCT
ABCA1	ATGTCCAGTCCAGTAATGGTTCTGT	CGAGATATGGTCCGGATTGC
DHCR24	CACTGTCTCACTACGTGTCGG	CCAGCCAATGGAGGTCAGC
ACAT1	GCAGCGAAGAGGCTCAATG	GCAGCATATACAGGAGCAATTGG
ABCG1	ATTCAGGGACCTTTCCTATTCGG	CTCACCACTATTGAACTTCCCG
LDLR	TCTGCAACATGGCTAGAGACT	TCCAAGCATTCGTTGGTCCC
ACAT2	GCGGACCATCATAGGTTCCTT	ACTGGCTTGTCTAACAGGATTCT
NPC1	GTCCAGCGCAGGTGTTTTC	GCCGAACATCACAACAGAGAC
CH25H	ATCACCACATACGTGGGCTTT	GTCAGGGTGGATCTTGTAGCG
NPC2	CAAAGGACAGTCTTACAGCGT	GGATAGGGCAGTTAATTCCACTC
CYP27A1	CGGCAACGGAGCTTAGAGG	GGCATAGCCTTGAACGAACAG
CYP46A1	TGTGTTTTTGGATTGGGCTAAGA	ACTCAGGACTCGTGACGATGA
CYP7B1	AAAACCACAAGTTGGGACACG	GAAGCTCAATGGGTATGTTGGAT
CD80	AAACTCGCATCTACTGGCAAA	GGTTCTTGTACTCGGGCCATA
ARG1	TCATCTGGGTGGATGCTCACAC	GAGAATCCTGGCACATCGGGAA
NOS	TTCAGTATCACAACCTCAGCAAG	TGGACCTGCAAGTTAAAATCCC
TNF-α	CCTCTCTCTAATCAGCCCTCTG	GAGGACCTGGGAGTAGATGAG
IL-1α	TGGTAGTAGCAACCAACGGGA	ACTTTGATTGAGGGCGTCATTC
IL-1β	ATGATGGCTTATTACAGTGGCAA	GTCGGAGATTCGTAGCTGGA
IL-6	ACTCACCTCTTCAGAACGAATTG	CCATCTTTGGAAGGTTCAGGTTG
TGF-β	GGCCAGATCCTGTCCAAGC	GTGGGTTTCCACCATTAGCAC
IL-10	TCAAGGCGCATGTGAACTCC	GATGTCAAACTCACTCATGGCT
CCL22	ATCGCCTACAGACTGCACTC	GACGGTAACGGACGTAATCAC
CCL24	ACATCATCCCTACGGGCTCT	CTTGGGGTCGCCACAGAAC
PPARγ	GGGATCAGCTCCGTGGATCT	TGCACTTTGGTACTCTTGAAGTT

Table S4. Detailed information of antibodies used in this study

Antibody	Manufacture	Catalog Number	RRID	Dilution
SLC25A1	Proteintech	15235-1-AP	AB_2254794	1:1000
iNOS	Affinity	AF0199	AB_2833391	1:1000
CD86	Proteintech	13395-1-AP	AB_2074882	1:1000
CD206	Proteintech	18704-1-AP	AB_10597232	WB 1:1000
				IHC 1:100
ARG1	Affinity	DF6657	AB_2838619	WB 1:1000

				IHC 1:100
p-STAT6	Affinity	AF3301	AB_2834720	1:1000
STAT6	Proteintech	51073-1-AP	AB_2197244	1:1000
PPARγ	Proteintech	16643-1-AP	AB_10596794	1:1000
Vimentin	Abcam	Ab92547	AB_10562134	1:50
Cytokeratin 7	Proteintech	66483-1-Ig	AB_2881849	1:50
HRP-conjugated Beta Actin Monoclonal antibody	Proteintech	HRP-66009	AB_2883836	1:10000
Goat Anti-Rabbit IgG H&L (FITC)	Abcam	Ab6717	AB_955238	1:200
Goat Anti-Mouse IgG H&L (Alexa Fluor® 647)	Abcam	Ab150115	AB_2687948	1:200

WB, western blotting; IHC, Immunohistochemistry.

Supplementary Figures

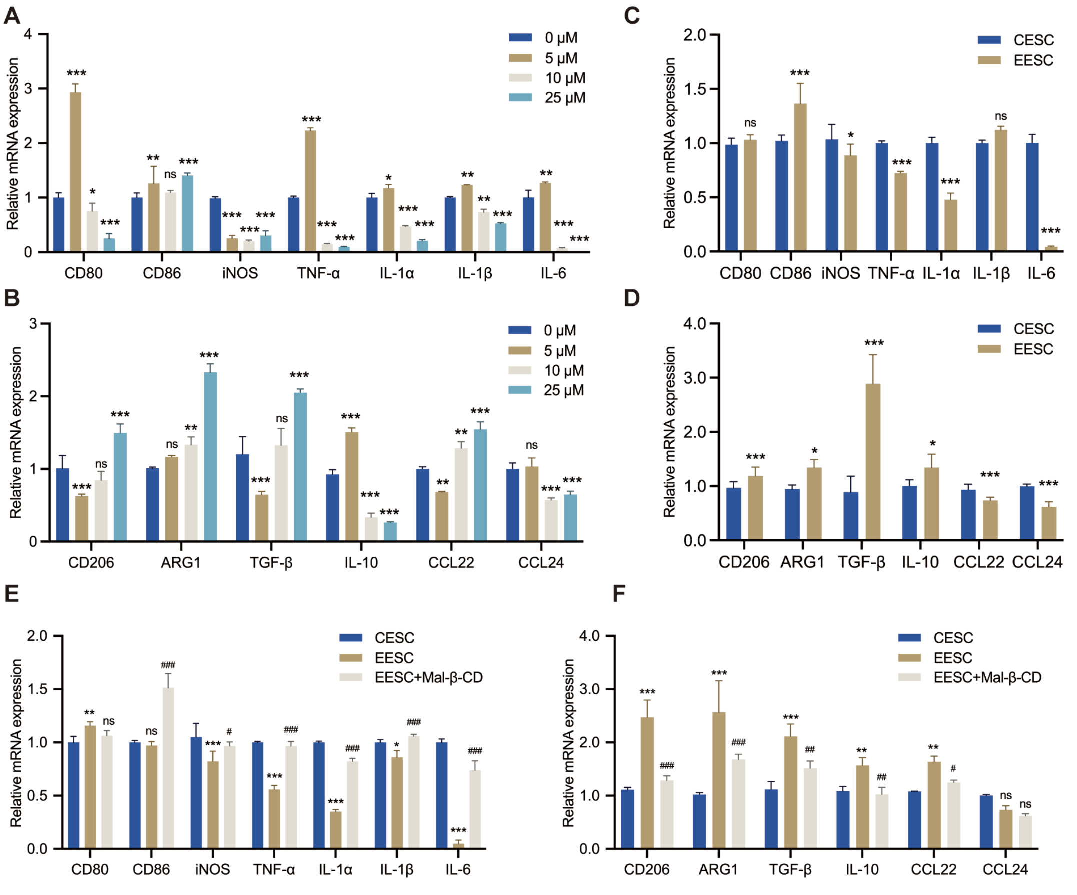

Figure S1. Cholesterol enhances the polarization of M2 phenotype

(A, B) The mRNA expression levels of M1 and M2 markers in control and cholesterol group (0, 5, 10, and 25 μM). (C, D) The mRNA expression levels of M1 and M2 markers in macrophages co-cultured with supernatants from CESCs and EESCs. (E, F) The mRNA expression levels of M1 and M2 markers in macrophages co-cultured with supernatants from CESCs, and EESCs (with or without Mal-β-CD). *

$P < 0.05$, ** $P < 0.01$, *** $P < 0.001$.

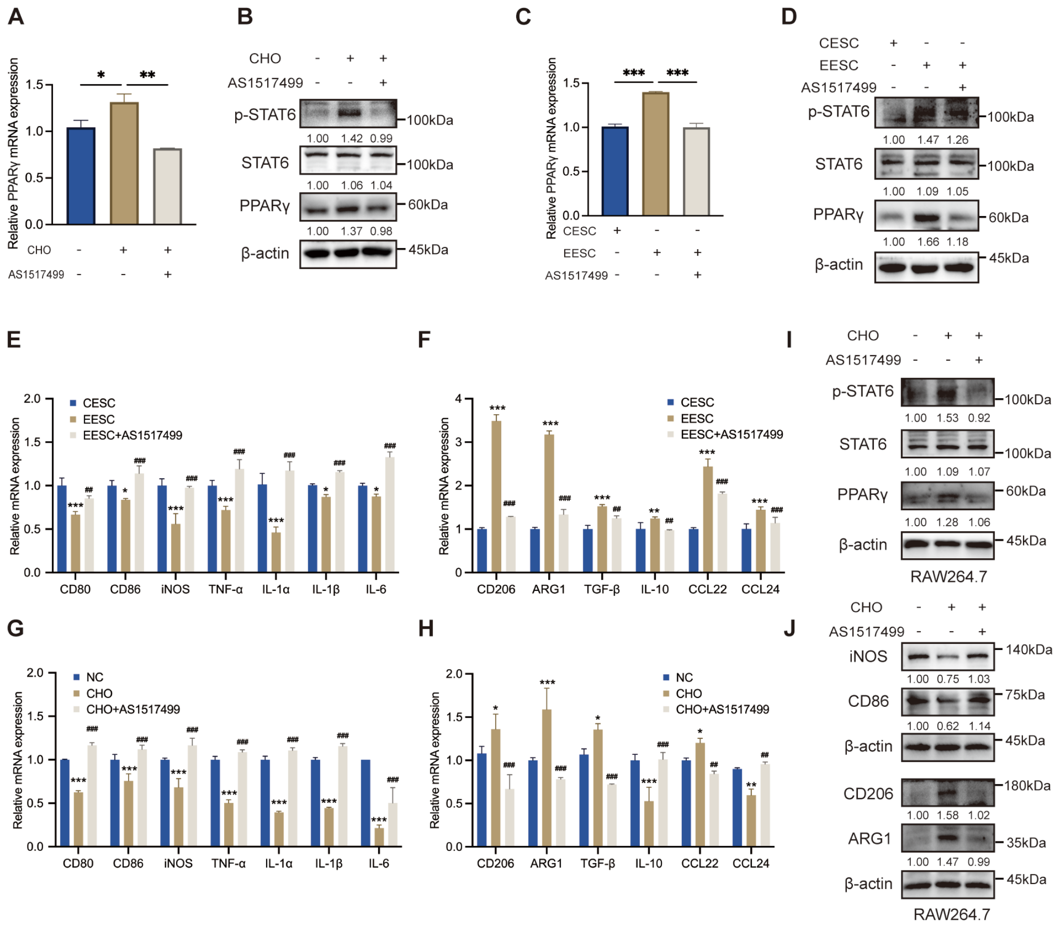

Figure S2. Cholesterol promotes M2 macrophage polarization through the STAT6/PPARγ pathway

(A, B) Changes in STAT6/PPARγ signaling activity in cholesterol treatment macrophages following pretreatment with 1 μM AS1517499 for 2 h. (C, D) Changes in STAT6/PPARγ signaling activity after co-culture with supernatants from CESCs or EESCs (with or without AS1517499 pretreatment). (E, F) The mRNA expression levels of M1 and M2 markers in THP-1-derived macrophages after co-culture with supernatants from CESCs or EESCs (with or without AS1517499 pretreatment). (G, H) The mRNA expression levels of M1 and M2 markers in THP-1-derived macrophages pretreated with/without AS1517499 and treated with cholesterol. (I) The protein levels of p-STAT6, STAT6 and PPARγ in RAW264.7 pretreated with/without AS1517499 and treated with cholesterol. (J) The protein levels of M1 and M2 markers in RAW264.7 pretreated with/without AS1517499 and treated with cholesterol. * $P < 0.05$, ** $P < 0.01$, *** $P < 0.001$.

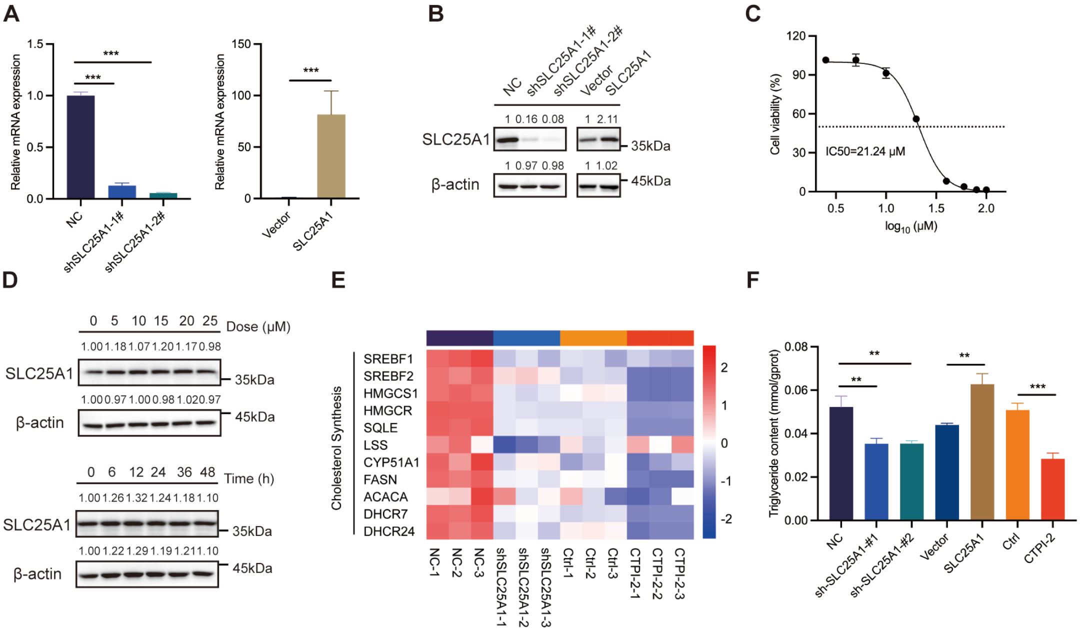

Figure S3. Construction of SLC25A1 cell lines

(A, B) The knockdown and overexpression efficiency of SLC25A1 in HESCs were confirmed by qRT-PCR and WB. (C) The IC50 (21.24μM) of CTPI-2 in HESCs for 24 h. (D) The protein expression of SLC25A1 after being treated with varying concentrations of CTPI-2 for different time periods. (E) Relative mRNA expression of cholesterol synthesis-related genes in the SLC25A1 knockdown and CTPI-2 treatment groups compared to those in the control groups based on RNA sequencing. (F) The triglycerides contents of supernatants from HESCs (shSLC25A1, OE-SLC25A1, CTPI-2 treatment groups and their respective control groups). $* P < 0.05$, $** P < 0.01$, $*** P < 0.001$.

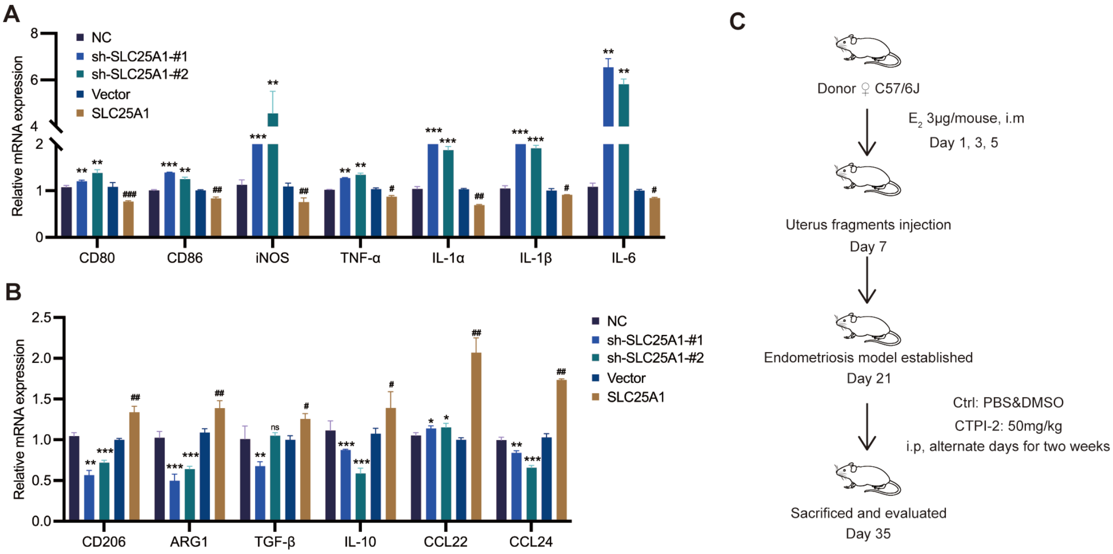

Figure S4. SLC25A1 promotes the M2-like macrophage polarization

(A, B) The mRNA expression levels of M1 and M2 markers on macrophages co-cultured with supernatants from HESCs of SLC25A1 knockdown and overexpression. (C) Flow chart of endometriosis animal model construction to detect the in vivo effects of SLC25A1. ∗ $P < 0.05$, ∗∗ $P < 0.01$, ∗∗∗ $P < 0.001$.

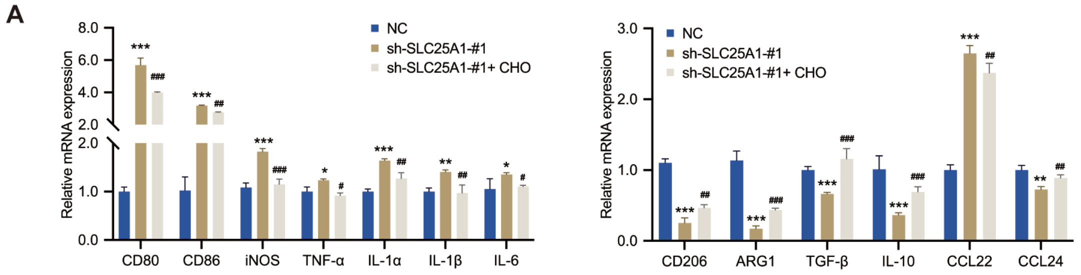

Figure S5. SCL25A1 regulates cholesterol metabolism in HESCs to promote M2 polarization of macrophages

(A) The mRNA expression levels of M1 and M2 markers on THP-1-derived macrophages co-cultured with supernatants from HESCs of SLC25A1 knockdown with or without cholesterol supplementation (10 μM, 48 h). ∗ $P < 0.05$, ∗∗ $P < 0.01$, ∗∗∗ $P < 0.001$.
